# The lncRNA HULC functions as an oncogene by targeting ATG7 and ITGB1 in epithelial ovarian carcinoma

**DOI:** 10.1038/cddis.2017.486

**Published:** 2017-10-12

**Authors:** Shuo Chen, Dan-Dan Wu, Xiu-Bo Sang, Li-Li Wang, Zhi-Hong Zong, Kai-Xuan Sun, Bo-Liang Liu, Yang Zhao

**Affiliations:** 1 Department of Gynecology, The First Affiliated Hospital of China Medical University, Shenyang, China; 2 Department of Biochemistry and Molecular Biology, College of Basic Medicine, China Medical University, Shenyang, China

## Abstract

Highly upregulated in liver cancer (HULC) is a long noncoding RNA (lncRNA), which has recently been identified as a key regulator in the progression of hepatocellular carcinoma, gliomas and gastric cancer. However, its role in epithelial ovarian carcinoma (EOC) remains unknown. In this study, HULC expression was examined in EOC, borderline and benign ovarian tumors, and normal ovarian tissues by RT-PCR. Ovarian cancer cell phenotypes, as well as autophagy-associated proteins were examined after HULC overexpression or downregulation by plasmid or small interfering RNA (siRNA) transfection, respectively. LncRNA–protein interactions were examined by ribonucleoprotein immunoprecipitation (RIP) assays. We found that HULC expression levels were higher in EOC tissues than normal samples. HULC overexpression induced cell proliferation, migration, invasion, whereas reduced cell apoptosis *in vitro* and induced tumor growth *in vivo*. In contrast, downregulation of HULC by siRNA transfection reduced cell proliferation, migration and invasion, and induced cell apoptosis and autophagy. Our results showed that HULC overexpression reduced ATG7, LC3-II and LAMP1 expression, while inducing SQSTM1 (P62) and ITGB1 expression. HULC downregulation had the opposite effects. Furthermore, RIP indicated that ATG7 interacted with HULC; ATG7 downregulation also induced cell proliferation, reduced apoptosis and inhibited autophagy *in vitro* by reducing LC3-II and LAMP1 expression, while inducing SQSTM1 expression. Furthermore, ATG7 co-transfection with HULC reversed the oncogenic effects of HULC both *in vitro* and *in vivo*; however, downregulating ATG7 did not affect cell migration and invasive ability. We found that ITGB1 siRNA co-transfection with HULC reversed the function of HULC in inducing ovarian cancer cell migration and invasive ability. Taken together, our results show that HULC may promote ovarian carcinoma tumorigenesis by inhibiting ATG7 and inducing progression by regulating ITGB1.

Epithelial ovarian cancer (EOC) is the second most common gynecologic malignancy in women worldwide.^1^ Although the optimization of current treatment modalities has improved outcomes in women with advanced disease over the past decade, the 5-year overall survival (OS) rate for this patient population remains at only 40%.^1, 2^ Ovarian cancer is disproportionately deadly because of the lack of sophisticated approaches for early diagnosis. Furthermore, tumorigenesis and progression of ovarian carcinoma are multistage processes. An increased understanding of the changes that occur in gene expression during carcinogenesis may result in improved diagnosis, treatment and prevention.

Cellular homeostasis involves maintaining a balance of multiple factors that contribute to a healthy cell environment. Changes in homeostasis may eventually lead to the development of cancer. A number of mechanisms exist to maintain cell homeostasis, including autophagy,^3, 4, 5, 6, 7^ apoptosis,^8, 9, 10^ cell cycle controls and cell aging. Recent studies have shown that, during caspase-mediated non-apoptotic cell death, the expression of autophagy-related genes, such as ATG5, ATG7 and Beclin-1, is upregulated.^11, 12^ This type of cell death is defined as type II programmed cell death, or autophagic cell death, and deregulation of this process can lead to changes in cell homeostasis, resulting in tumorigenesis.^13^ Therefore, it is essential to determine the underlying molecular mechanisms of autophagy in ovarian cancer, which may be helpful for early detection, diagnosis and treatment.

Long noncoding RNAs (lncRNAs) are novel regulators of gene expression involved in the regulation of many cellular processes including tumor growth and development, apoptosis, proliferation, differentiation and cell autophagy, and, therefore, are implicated in cancers and other diseases.^14, 15, 16^ The highly upregulated in liver cancer (HULC) human lncRNA is multifunctional and has been implicated in various cellular processes. Upregulation of HULC has been detected in many human malignancies, such as hepatocellular carcinoma,^17^ esophageal cancer,^18^ osteosarcoma,^19^ pancreatic cancer,^20^ gliomas^21^ and gastric cancer.^22^ However, its role in ovarian cancer and its relationship with autophagy remain elusive. In this study, we demonstrate that HULC may promote ovarian carcinoma tumorigenesis and progression, and inhibit autophagy.

## Results

### HULC expression correlates with the pathogenesis of ovarian carcinoma

**Figure 1:**
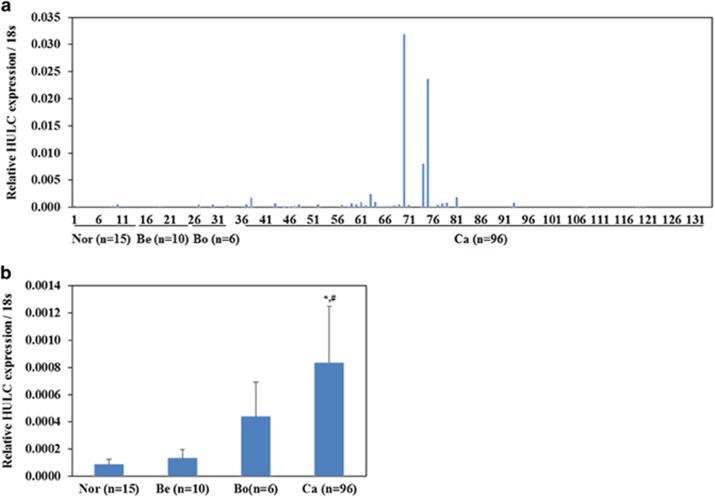
Correlation of lncRNA HULC expression with pathogenesis of ovarian carcinoma. HULC expression in ovarian cancer tissues was higher than borderline ovarian tumors, benign ovarian tumors and normal ovarian tissues (**a**). HULC expression was significantly higher in EOCs than normal ovarian tissues and benign ovarian tumors (**b**). *versus normal ovarian tissues; ^#^*versus* benign tumors; *P*<0.05

The expression levels of HULC were examined in EOC, borderline and benign ovarian tumors and in normal ovarian tissues by RT-PCR (Figure 1a). HULC expression levels were higher in EOC tissues than in the normal samples and benign ovarian tumors (*P*<0.05; Figure 1b).

### HULC overexpression induces proliferation of ovarian carcinoma cells

**Figure 2:**
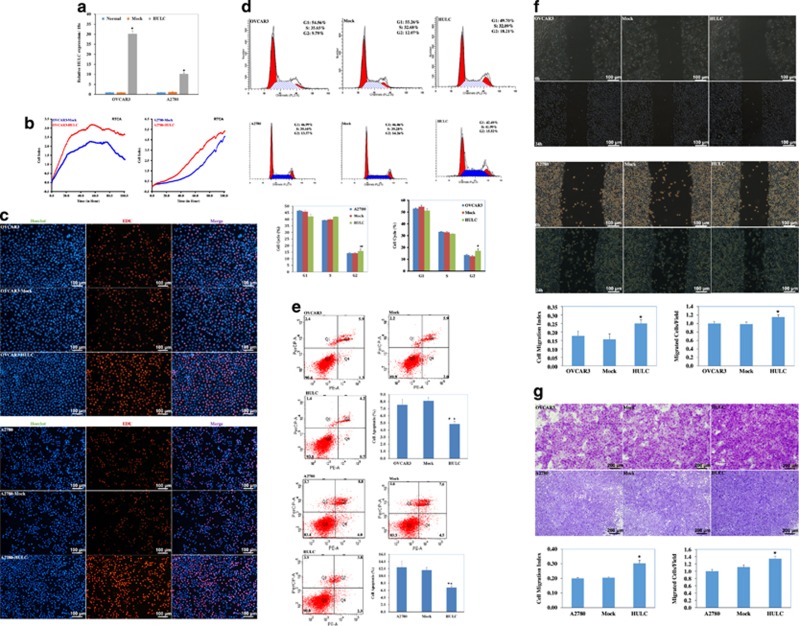
HULC overexpression induces ovarian carcinoma cell proliferation, migration and invasion ability. HULC overexpression (**a**) induced cell proliferation compared with mock-transfected cells by RTCA (**b**), EDU (**c**) and cell cycle (**d**) assay. HULC overexpression reduced cell apoptosis (**e**), induced cell migration ability (**f**) and invasion ability compared with control and mock-transfected cells (**g**). Results are representative of three separate experiments; data are expressed as the mean±S.D., *P<0.05

Analysis by qRT-PCR indicated that HULC plasmid transfection increased lncRNA HULC expression (*P*<0.05; Figure 2a). The real-time cell analyzer (RTCA) assay showed that viability of HULC-transfected cells was increased as compared with mock-transfected cells (*P*<0.05; Figure 2b). EdU staining demonstrated significantly upregulated proliferation in cells overexpressing HULC (Figure 2c). Cell cycle assays showed that HULC overexpression induced S/G_2_ progression as compared with the control and mock-transfected cells (*P*<0.05; Figure 2d). Apoptosis assays showed that HULC overexpression reduced apoptosis as compared with the control and mock-transfected cells (*P*<0.05; Figure 2e).

### HULC overexpression induces ovarian carcinoma cell migration and invasion

Wound-healing assays showed that HULC overexpression induced cell migration ability (*P*<0.05; Figure 2f). Transwell assays showed that cells transfected with HULC possessed increased invasion ability (*P*<0.05; Figure 2g), as compared with control and mock-transfected cells.

### Downregulation of HULC suppresses ovarian carcinoma cell proliferation

**Figure 3:**
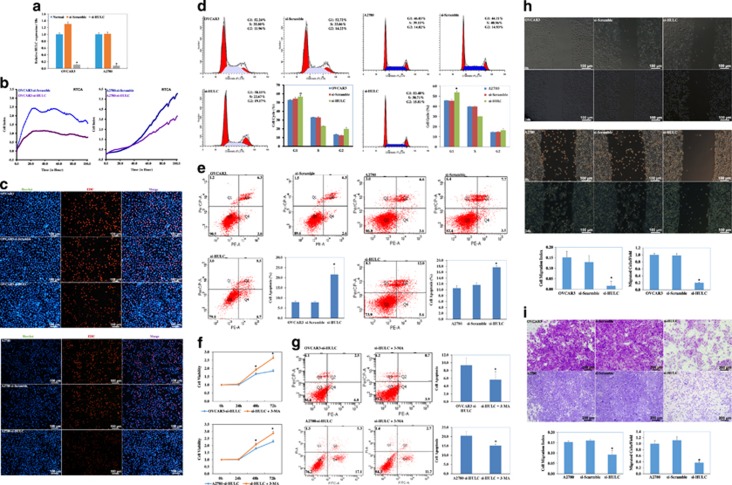
HULC downregulation suppresses ovarian carcinoma cell proliferation, migration and invasion ability. QRT-PCR analysis indicated that HULC siRNA transfection reduced the expression of HULC (**a**). HULC downregulation by HULC siRNA transfection reduced cell proliferation compared with mock-transfected cells by RTCA (**b**), EDU (**c**) and cell cycle assay (**d**), while inducing cell apoptosis (**e**), 3-MA could reduce si-HULC’s function in inhibiting cell proliferation (**f**) and inducing cell apoptosis (**g**). HULC downregulation reduced cell migration (**h**) and invasion (**i**) ability compared with the control and mock-transfected cells. Results are representative of three separate experiments; data are expressed as the mean±S.D., *P<0.05

Analysis by qRT-PCR indicated that transfection of small interfering RNA (siRNA) targeting HULC reduced the expression of HULC (*P*<0.05; Figure 3a). Our RTCA assays indicated that the viability of si-HULC-transfected cells was continuously lower than mock-transfected cells (*P*<0.05; Figure 3b). EdU staining demonstrated significantly reduced cell proliferation capacity in si-HULC-transfected cells (Figure 3c). Cell cycle assays showed that HULC downregulation induced G_1_/S arrest as compared with the control and mock-transfected cells (*P*<0.05; Figure 3d). Apoptosis assays showed that HULC downregulation induced apoptosis as compared with the control and mock-transfected cells (*P*<0.05; Figure 3e), and that the autophagy inhibitor 3-MA could reduce si-HULC’s function in inhibiting cell proliferation (*P*<0.05; Figure 3f) and inducing cell apoptosis (*P*<0.05; Figure 3g).

### HULC downregulation reduces ovarian carcinoma cell migration and invasion

Wound-healing assay showed that HULC downregulation reduced cell migration compared with the control and mock-transfected cells (*P*<0.05; Figure 3h). Transwell assays showed that cells transfected with si-HULC had reduced invasion ability compared with control and mock-transfected cells (*P*<0.05; Figure 3i).

### HULC overexpression regulates ATG7, LC3, SQSTM1 (P62), LAMP1 and ITGB1 mRNA and protein expression

**Figure 4:**
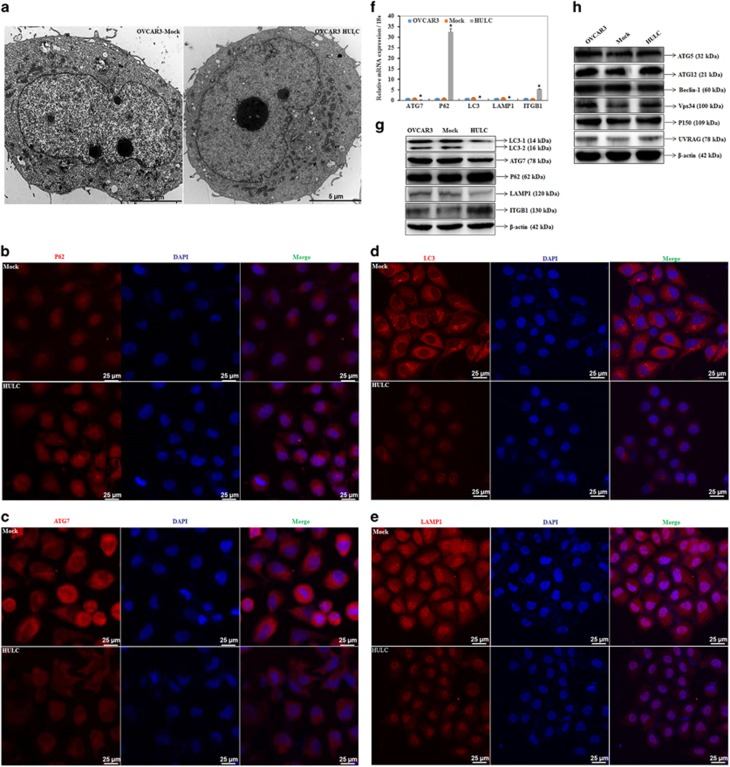
HULC overexpression regulates ATG7, LC3, SQSTM1 (P62), LAMP1 and ITGB1 mRNA or protein expression. HULC overexpression induced mitochondria formation (**a**), induced SQSTM1 expression (**b**), inhibited ATG7 (**c**), LC3 (**d**) and LAMP1 expression (**e**). The mRNA (**f**) and protein (**g**) expression of ATG7, LC3 and LAMP1 were significantly lower than in the control, whereas expression of SQSTM1 and ITGB1 was significantly increased compared with the negative control. No significant differences were with ATG5, Beclin-1, ATG12, Vps34, P150 and UVRAG expression (**h**). *P<0.05

We observed induced mitochondria formation in cells transfected with HULC by transmission electron microscopy (Figure 4a). Results from immunofluorescence assays indicated that HULC overexpression induced expression of SQSTM1 (Figure 4b), while inhibiting ATG7 (Figure 4c), LC3 (Figure 4d) and LAMP1 (Figure 4e) expression. We performed qRT-PCR and western blot analysis to measure ATG7, LC3, SQSTM1, LAMP1 and ITGB1 mRNA or protein expression levels after HULC overexpression in OVCAR3 cells. Both mRNA and protein expression of ATG7, LC3 and LAMP1 were significantly lower than in the control, whereas expression of SQSTM1 and ITGB1 was significantly increased compared with the negative control (*P*<0.05; Figures 4f and g). However, there were no significant differences in ATG5, Beclin-1, ATG12, Vps34, P150 and UVRAG expression (Figure 4h).

### HULC downregulation regulates ATG7, LC3, SQSTM1 (P62), LAMP1 and ITGB1 mRNA and protein expression

**Figure 5:**
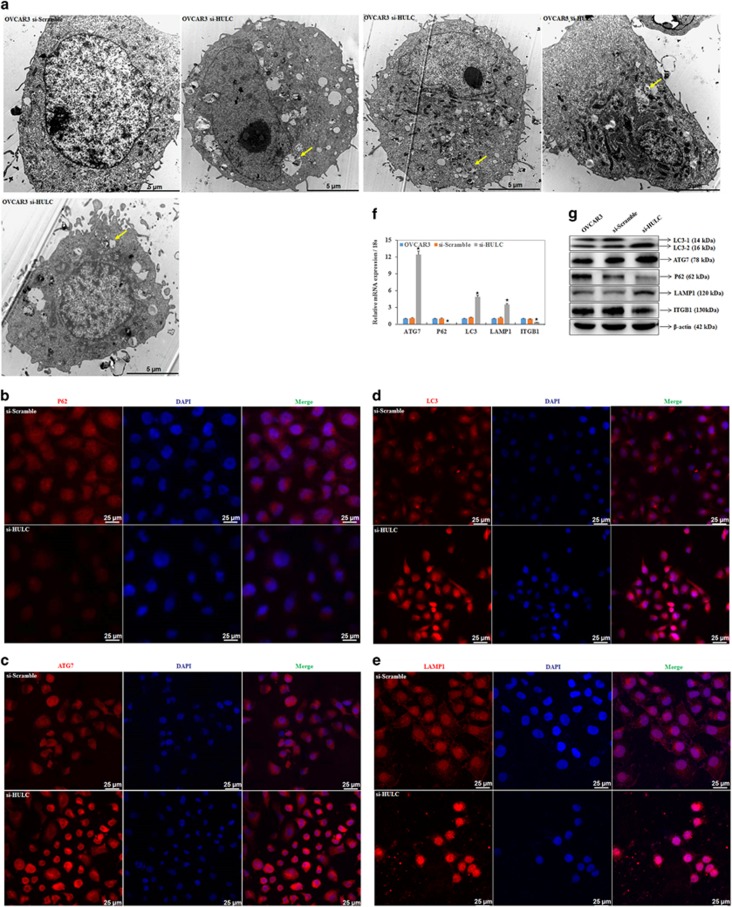
HULC downregulation regulates ATG7, LC3, SQSTM1 (P62), LAMP1 and ITGB1 mRNA or protein expression. HULC downregulation induced autophagosomes formation (**a**), reduced SQSTM1 expression (**b**), induced ATG7 (**c**), LC3 (**d**) and LAMP1 expression (**e**). The mRNA (**f**) and protein (**g**) expression of ATG7, LC3 and LAMP1 were significantly higher than in the control, whereas expression of SQSTM1 and ITGB1 was significantly decreased compared with the negative control. *P<0.05

We observed autophagosome formation in cells transfected with si-HULC by transmission electron microscopy (Figure 5a). Immunofluorescent (IF) results indicated that HULC downregulation inhibited SQSTM1 expression (Figure 5b), while inducing ATG7 (Figure 5c), LC3 (Figure 5d) and LAMP1 expression (Figure 5e). Following HULC siRNA transfection of ovarian cancer cells, ATG7, LC3 and LAMP1 mRNA and protein expression both were significantly higher than in the control, whereas expression of SQSTM1 and ITGB1 was significantly decreased by HULC siRNA transfection compared with the negative control (*P*<0.05; Figures 5f and g).

### HULC overexpression induces ovarian carcinoma cell tumorigenesis *in vivo*

**Figure 6:**
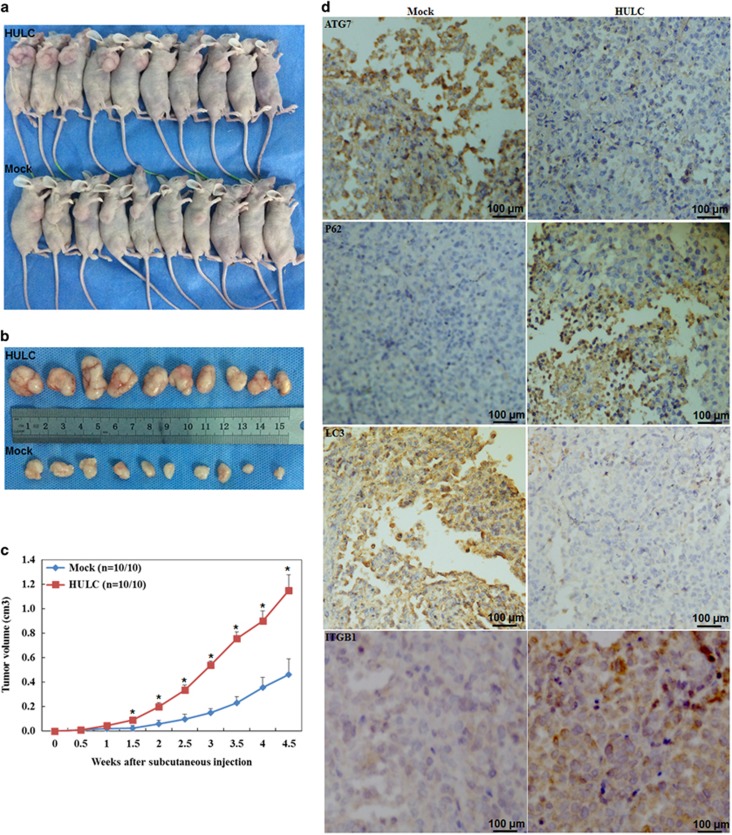
HULC overexpression induces the tumorigenicity of ovarian carcinoma cells in *vivo*. A2780 cells with HULC overexpression induced tumorigenicity after inoculation (**a**) and exhibited bigger tumor volume (**b** and **c**). HULC overexpression reduced ATG7 and LC3-II expression, induced SQSTM1 and ITGB1 expression (**d**)

In our study, mice injected with A2780-HULC overexpression cells showed a significantly higher rate of tumorigenicity after inoculation compared with the control group (*P*<0.05; Figure 6c), and exhibited larger tumor volumes during the same observation period (Figures 6a and b). Immunohistochemistry (IHC) analysis showed significantly lower ATG7 and LC3 expression, while SQSTM1 and ITGB1 expression was upregulated in the HULC overexpression group compared with the control group (Figure 6d).

### HULC co-immunoprecipitates with ATG7

**Figure 7:**
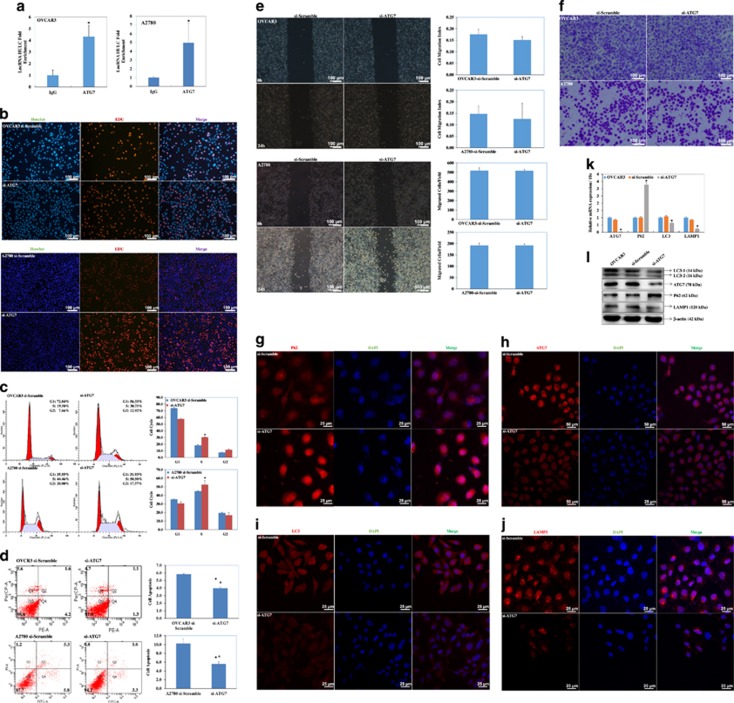
HULC co-immunoprecipitates with ATG7. RIPs assay demonstrated an enrichment of lncRNA HULC (**a**). Si-ATG7 transfection induced cell proliferation (**b**), S/G2 progression (**c**), reduced cell apoptosis (**d**). Si-ATG7 transfection had no effect in migration and invasion ability (**e** and **f**). Si-ATG7 transfection induced SQSTM1 expression (**g**), inhibited ATG7 (**h**), LC3 (**i**) and LAMP1 expression (**j**) by Immunofluorescence. The mRNA (**k**) and protein (**l**) expression of ATG7, LC3 and LAMP1 were significantly lower than in the control, whereas expression of SQSTM1 was significantly increased compared with the negative control. *P<0.05

Ribonucleoprotein immunoprecipitation (RIP) assays were performed to detect interactions between RNA and the autophagic protein ATG7. RNA obtained from RIP using an antibody against ATG7 was subjected to qPCR analysis in both A2780 and OVCAR3 cells, which demonstrated an enrichment of the lncRNA HULC (Figure 7a).

### ATG7 downregulation induces ovarian carcinoma cell proliferation

EdU staining demonstrated ATG7 siRNA transfection significantly upregulated proliferation (Figure 7b). In addition, ATG7 downregulation induced S/G_2_ progression (*P*<0.05; Figure 7c), and reduced apoptosis (*P*<0.05; Figure 7d) as compared with the mock-transfected cells. However, ATG7 downregulation did not affect cell migration and invasion (*P*>0.05; Figures 7e and f).

### ATG7 downregulation regulates ATG7, LC3, SQSTM1 (P62) and LAMP1 mRNA and protein expression

The immunofluorescence assays indicated that si-ATG7 transfection induced SQSTM1 expression (Figure 7g), while inhibiting ATG7 (Figure 7h), LC3 (Figure 7i) and LAMP1 (Figure 7j) expression. We used qRT-PCR and western blotting to determine ATG7, LC3, SQSTM1 and LAMP1 mRNA or protein expression levels after si-ATG7 transfection in OVCAR3 cells. Both mRNA and protein expression of ATG7, LC3 and LAMP1 were significantly lower, whereas SQSTM1 (P62) expression was increased as compared with the negative control (Figures 7k and l).

### ATG7 co-transfection with HULC reverses HULC oncogenic effects

**Figure 8:**
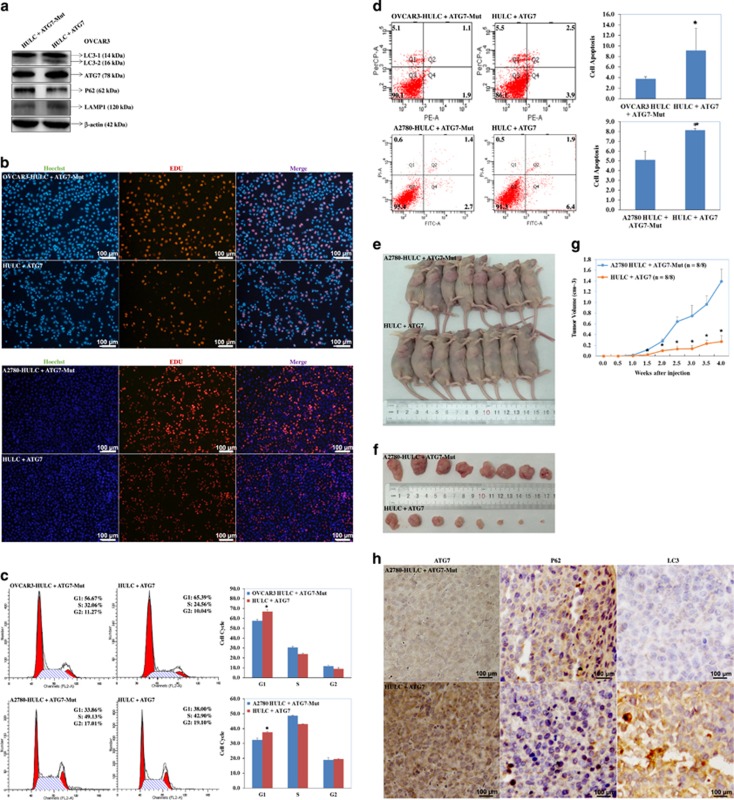
ATG7 co-transfection with HULC reverses HULC oncogenic effects both *in* *vivo* and *in* *vitro*. Compared with A2780-HULC overexpression cells, the co-transfection of ATG7 and HULC induced ATG7, LC3-II and LAMP1 expression, reduced SQSTM1 expression (**a**), reduced cell proliferation (**b**), induced G_1_ arrest (**c**) and apoptosis (**d**), and showed significantly smaller tumor volumes (**e** and **f**) and lower rates of tumorigenicity *in vivo* (**g**). IHC analysis showed higher ATG7 and LC3 expression, whereas SQSTM1 expression was downregulated in the ATG7 and HULC co-transfection group (**h**)

The western blotting results indicated that co-transfection of ATG7 and HULC induced ATG7, LC3-II, LAMP1 expression, whereas reduced SQSTM1 expression (Figure 8a), reduced cell proliferation (Figure 8b), induced G_1_ arrest (*P*<0.05; Figure 8c) and apoptosis (*P*<0.05; Figure 8d) as compared with HULC-transfected cells.

### ATG7 co-transfection with HULC reverses HULC oncogenic effects *in vivo*

Mice injected with A2780 cells co-transfected with ATG7 and HULC showed significantly lower rates of tumorigenicity after inoculation as compared with the HULC-ATG7-Mut group (*P*<0.05; Figure 8g) and exhibited smaller tumor volumes during the same observation period (Figures 8e and f). IHC analysis showed higher ATG7 and LC3 expression, whereas SQSTM1 expression was downregulated in the ATG7 and HULC co-transfection group as compared with the HULC-ATG7-Mut group (Figure 8h).

### Si-ITGB1 co-transfection with HULC reverses HULC’s effect in inducing migration and invasion

**Figure 9:**
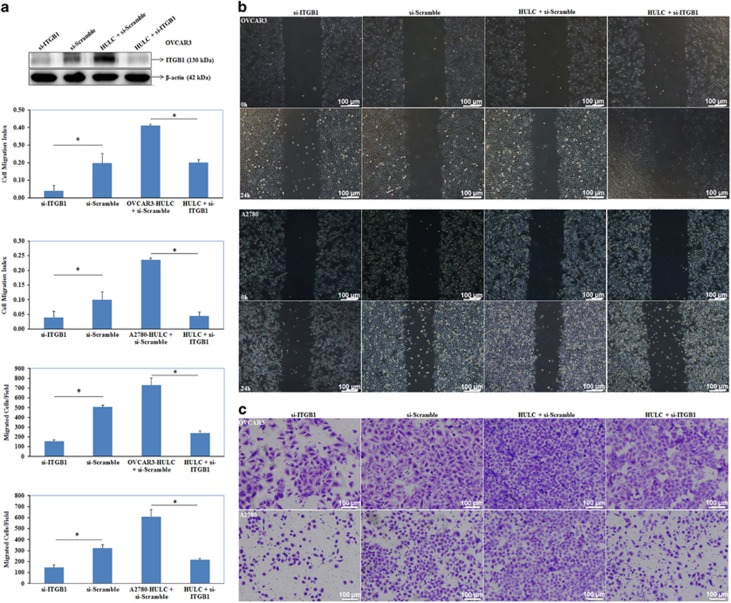
si-ITGB1 co-transfection with HULC reverses HULC’s effect in inducing migration and invasion. Compared with HULC overexpression group, si-ITGB1 co-transfection with HULC reduced ITGB1 expression (**a**), inhibited migration (**b**) and invasion ability (**c**). Results are representative of three separate experiments; data are expressed as the mean±S.D., *P<0.05

Western blotting results showed that si-ITGB1 co-transfection with HULC reduced ITGB1 expression as compared with the HULC-si-Scramble group (Figure 9a). Besides, si-ITGB1 co-transfection with HULC inhibited the tumor-promoting effect of HULC by inhibiting tumor metastasis (*P*<0.05, Figure 9b) and invasion (*P*<0.05, Figure 9c).

## Discussion

Studies have demonstrated that the lncRNA HULC is associated with the proliferation, invasion, metastasis and survival of tumor cells in certain cancers.^17, 18, 19, 20, 21, 22^ Furthermore, overexpression of HULC serves as an independent indicator of patient prognosis, by predicting the rates of recurrence and disease-free survival.^20^ Our results showed that HULC expression was significantly higher in EOC than in benign tumor, and normal ovarian tissues. Our results are consistent with Peng *et al.*^23^, who showed that the lncRNA HULC is a novel biomarker in patients with pancreatic cancer and diffuse large B-cell lymphoma.^24^ This indicates that HULC may have potential as a biomarker in EOC diagnosis.

HULC is known to induce proliferation, migration and invasion in cell culture.^21^ We confirmed this observation by showing that the overexpression of HULC *in vitro* induced cell proliferation, migration, invasion and reduced cell apoptosis, whereas HULC downregulation by siRNA transfection reduced cell proliferation, migration, invasion and induced cell apoptosis, which suggests that HULC may functions as an oncogene in EOC. Furthermore, through electron microscopy, we found that HULC increased mitochondria formation, while si-HULC transfection induced autophagosome; we also found that adding the autophagy inhibitor 3-MA reversed the effects of si-HULC in inducing apoptosis and inhibiting cell proliferation, suggesting than HULC may promote tumorigenesis by inhibiting autophagy.

Autophagy involves numerous steps: initiation, nucleation, elongation, closure of the membranes that form the autophagosome, fusion with the lysosome and the recycling of macromolecular precursors. Specific autophagy-related proteins regulate each step. Two ubiquitin-like conjugation systems elongate the autophagosome membrane. The ubiquitin-like protein ATG12 is conjugated to ATG5 in a process requiring the E1-like enzyme ATG7. A similar lipid conjugation system (also using ATG7) attaches phosphatidylethanolamine (PE) to the microtubule-associated protein 1 light chain 3 (MAP1LC3) and GABA type A receptor-associated protein (GABARAP) protein families. Furthermore, Beclin-1 (BCL-2-interacting moesin-like coiled-coil protein 1), its signaling complex P150, VPS34 (class III phosphoinositide-3-kinase) and ultraviolet irradiation resistant-associated gene (UVRAG) are all responsible for vesicle nucleation of the phagophore membrane.^25, 26^ LC3 was originally identified as a subunit of microtubule-associated proteins 1A and 1B and was subsequently found to be similar to the yeast protein Atg8/Aut7/Cvt5, which is critical for autophagy.^24, 27^ The conversion of LC3 to the lower migrating form, LC3-II, has been used as an indicator of autophagy.^28, 29, 30^ SQSTM1 (P62), has been implicated as a potential oncogene in other settings, including human hepatocellular carcinomas,^31^ lung carcinomas,^32^ pancreatic carcinomas, breast carcinomas,^33, 34^ prostate cancer^35^ and in immortalized baby mouse kidney cells.^36^ Recently, studies have shown that its accumulation represents a block to autophagosome clearance, and it has been well studied as a negative regulator of autophagy.^37, 38, 39, 40, 41, 42, 43, 44^ It can also be conjugated to LC3, participating in autophagy. Studies have shown that when LC3-II is upregulated, SQSTM1 (P62) is reduced, indicating that the autophagy is progressing, otherwise the autophagy flow is blocked.^45, 46^ Following ATG7 knockdown, inhibition of autophagy was verified by LC3-II downregulation and overexpression of the autophagy substrate SQSTM1/P62.^47^ Researchers have also shown that LAMP1, a lysosome surface marker, could be used for detecting the combination of autophagy and lysosomes.

Our results showed that HULC overexpression reduced ATG7, LC3-II and LAMP1 expression, while inducing SQSTM1 expression. In contrast, HULC downregulation led to ATG7, LC3, LAMP1 overexpression and SQSTM1 inhibition. Furthermore, HULC overexpression *in vivo* induced tumor formation, and reduced ATG7, LC3 expression, while inducing SQSTM1 expression; however, we found that HULC overexpression did not influence ATG5, ATG12, Beclin-1, VPS34, P150 or UVRAG expression. Therefore, we suggest that the lncRNA HULC may cause changes in cell homeostasis by inhibiting autophagy and promoting ovarian cancer by regulating ATG7, LC3, LAMP1 and SQSTM1 expression.

LncRNAs frequently function both in cis (at the site of their transcription), as well as in trans (at sites on other chromosomes), which highlights potential functions as interfaces with the epigenetic machinery, roles in chromatin organization and regulation of gene expression. The biogenesis of many lncRNAs is similar to mRNAs.^48^ It is currently believed that lncRNAs conduct their regulatory functions in the form of RNA–protein complexes through interactions with chromatin-modifying complexes and regulation of gene expression. Our RIP results showed that ATG7, but not LC3, SQSTM1 or LAMP1 could interact with HULC. Our results showed that ATG7 downregulation could also induce ovarian cancer cell proliferation, reduce apoptosis by reducing LC3-II and LAMP1 expression, and induce SQSTM1 expression; furthermore, ATG7 co-transfection with HULC partly reversed the function of HULC in tumorigenesis both *in vitro* and *in vivo*. This suggests that HULC may combine with ATG7 and inhibit the ATG7 pathway. Therefore, we suggest that HULC may function as an oncogene and autophagy inhibitor through inhibiting ATG7 in EOC. However, dysfunction of ATG7 does not make sense in ovarian cancer migration and invasion.

Integrins are a large family of cell surface adhesion proteins that are involved in epithelial cell–matrix interactions. The upregulation of integrins is associated with malignancy, particularly during invasion, metastasis and angiogenesis. Increasing evidence suggests that ITGB1 is frequently upregulated in ovarian cancer, and promotes ovarian tumorigenesis and cancer progression.^49^ We found that HULC overexpression induces ITGB1 expression, while si-HULC overexpression had the opposite effect. Moreover, si-ITGB1 co-transfection with HULC inhibited the tumor-promoting effect of HULC by inhibiting tumor metastasis and invasion, suggesting that HULC may promote ovarian cancer progression by regulating ITGB1.

The objective of this study demonstrates that HULC may promote ovarian carcinoma tumorigenesis by inhibiting ATG7 and induce progression by regulating ITGB1. We suggest that HULC may be an important diagnostic marker and potential therapeutic target in EOC. The inhibition of HULC expression may prove to be an effective genetic therapeutic strategy for EOC. However, further investigation will be required to elucidate the specific molecular mechanisms involved and to identify potential clinical applications of HULC in the treatment of epithelial ovarian carcinoma (EOC).

## Materials and methods

### EOC specimens

EOC tissues, borderline tumor tissues, benign tumor tissues and normal ovarian tissue specimens were collected from patients who had undergone surgical resection at the Department of Gynaecology of the First Affiliated Hospital of China Medical University (Shenyang, Liaoning, China). The tumor specimens were independently confirmed by two pathologists. None of the patients had preoperative chemotherapy or radiotherapy. Informed written consent was obtained from all participants and the research protocol was approved by the China Medical University Ethics Committee (no: 2014-27).

### Cell culture and transfection

The A2780 human ovarian carcinoma cell lines were cultured in Dulbecco’s modified Eagle's medium (HyClone, Logan, UT, USA), and OVCAR3 cells were cultured in RPMI-1640 (HyClone) supplemented with penicillin/streptomycin (100 U/ml) with 10% fetal bovine serum (FBS) at 5% CO_2_ and 37 °C. HULC plasmid (5′-CTCGAGATGGGGGTGGAACTCATGATGGAATTGGAGCCTTTACAAGGGAATGAAGAGACAAGAGCTCTCTTTATGCCACGTGAGGATACAGCAAGGCCCCAATCTGCAAGCCAGGAAGAGTCGTCACGAGAACCAGACCATGCAGGAACTCTGATCGTGGACATTTCAACCTCCAGAACTGTGATCCAAAATGCATATGTATCTTTGGAAGAAACTCTGAAGTAAAGGCCGGAATATTCTTTGTTTAAAACATTAAAAACAAAACAGACCAAAGCATCAAGCAAGAAGTTTCCTGGCAATAAACTAAGCACAGCATTATTTTTTAAGGAACACAAATTAAGTGTTCAACCTGTGGCAAATTTGTACTTTCTCCCTGAATTATGTTGTTATCAAAGAAAAAAATTGGGAAGCATGGCAAAATATCATCAAAACTGAAACTAGAATTAAACAAAACTAAATTAAAATGAAATAAAATGATGTCCATTCTTAAGGTACC-3′), HULC siRNA (sense: 5′-GGAAGAAACUCUGAAGUAAdtdt-3′ anti-sense: 5′-UUACUUCAGAGUUUCUUCCdtdt-3′), ATG7 siRNA (sense: 5′-GAGAUAUGGGAAUCCAUAAdTdT-3′ anti-sense: 5′-UUAUGGAUUCCCAUAUCUCdTdT-3′), and ITGB1 siRNA (sense: 5′-CUGUUCUUUGGAUACUAGUdTdT-3′ anti-sense: 5′-ACUAGUAUCCAAAGAACAGdTdT-3′) transfections were carried out using Lipofectamine 2000 according to the manufacturer's instructions.

### Real-time cell analyzer

For real-time cell proliferation assays, 50 *μ*l medium was added to each well of a 96-well E-plate for establishment of background levels. Subsequently, 5 × 10^3^ cells in 100 *μ*l medium were seeded per well into the E-plate. After incubation at room temperature for 30 min, the E-plates containing cells were placed on the RTCA SP/MP station positioned in a cell culture incubator. The CI values were measured automatically every 15 min (up to 99 h) to obtain a continuous proliferation curve.

### Cell cycle analysis

Cells were fixed with 70% ice-cold ethanol in −20 °C overnight, washed with phosphate-buffered saline (PBS), and then stained with PI (contain RNAase) following the manufacturer’s protocol (BD Biosciences, San Jose, CA, USA). The PI signal was examined by a flow cytometry; a total of 10 000 cells were assessed for each sample.

### Apoptosis assay

Apoptosis was quantified using 7-AAD/PI staining and flow cytometry with PE/FITC-labeled annexin V (BD Pharmingen, San Diego, CA, USA) following the manufacturer’s protocol and flow cytometry. Cells were collected 48 h after transfection, washed twice with cold PBS, and resuspended at 1 × 10^6^ cells/ml and mixed with 100 *μ*l of 1 × buffer and 5 *μ*l Annexin V-PE/FITC and 7-AAD/PI, incubated for 15 min in the dark, 400 *μ*l 1 × buffer was added, and the cells were subjected to cytometry flow within 1 h.

### Wound-healing assay

Cells were cultured to 80% confluence in a six-well culture dish. After scratching with a 200 *μ*l pipette tip, cells were washed with PBS and cultured in FBS-free medium. Wounds were observed by microscopy and photographed at 0 and 24 h. The wound areas were measured using Image J software (National Institutes of Health, Bethesda, MD, USA). The rate of wound healing was calculated as: area of original wound − area of actual wound at different times)/area of original wound × 100%.

### Invasion assay

Matrigel-coated transwell cell culture chambers (BD Biosciences) were used for the invasion assays. Filters were coated with 30 *μ*l of basement membrane Matrigel at a dilution of 1 : 15. Cells (5 × 10^4^) resuspended in 200 *μ*l of serum-free medium were layered in the upper compartment of the transwell inserts. The bottom chambers contained 600 *μ*l of complete medium serving as the chemoattractant. After incubation for 24 h at 37 °C, cells on the upper surface of the filter were removed using a cotton swab and the invaded cells at the bottom of the upper chamber were fixed with formaldehyde, stained with crystal violet, and counted under an Olympus fluorescence microscope (Tokyo, Japan).

### Autophagolysosome detection by transmission electron microscopy

Cells were fixed in 0.2 % glutaraldehyde in PBS (pH 7.4) for 2 h at room temperature, postfixed in 1 % osmium tetroxide in water for 1 h and then stained in 2 % uranyl acetate in water for 1 h in the dark. After dehydration in an ascending series of ethanol, the samples were embedded in Durcupan ACM for 6 h, and cut into 80 nm sections. These sections were stained with uranyl acetate and lead citrate, and examined with a transmission electron microscope (Philips CM, Amsterdam, The Netherlands).

### Real-time RT-PCR

Total RNA was isolated from ovarian cancer cell lines or tissues using TRIzol Reagent (Takara,, Shiga, Japan) and was reverse transcribed to cDNA using an avian myeloblastosis virus reverse transcriptase and random primers (Supplementary Table 1) according to the manufacturer’s protocol. The cDNA was then amplified by real-time quantitative PCR using a SYBR Premix Ex Taq II kit (Takara). Expression levels of each target gene was normalized to 18S mRNA. The data analysis was performed based on the sample threshold cycle (Ct) value from three independent experiments.

### Western blotting

Cells were harvested and lysed in RIPA buffer containing protease inhibitors and the total cell proteome collected. Proteins were separated by 7.5%, 10% or 15% SDS-polyacrylamide gel electrophoresis and electrotransferred to Hybond membranes (Amersham, Munich, Germany). Fat-free milk (5%) was used to block membranes for 2 h at room temperature. After blocking, primary antibodies targeting ATG7, SQSTM1, LAMP1, Beclin-1, ATG12 (1 : 1000, Proteintech Group, USA), LC3 (1 : 2000, MBL, Woburn, MA, USA), ITGB1, ATG5, Vps34, p150, UVRAG (1 : 1000, Abclonal, Boston, MA, USA) and *β*-actin (1:3000, Proteintech Group, Chicago, IL, USA) were incubated with the blot overnight at 4 °C. The following day, secondary antibodies were added for 2 h at room temperature after the membrane was washed three times with TBST. Protein was visualized using an enhanced chemiluminescence system according to the manufacturer’s protocol (Santa Cruz Biotechnology, California, CA, USA).

### Nude mouse xenograft assay

All animal experiments were undertaken in accordance with the National Institutes of Health Guide for the Care and Use of Laboratory Animals, with the approval of the China Medical University Animal Care and Use Committee. Female BALB/c nude mice, 4 weeks old were obtained from Vital River Laboratories (Beijing, China) and were routinely housed in rooms that were temperature- and light-controlled (12-h dark/12-h light). Animals had free access to food and water. A total of 1 × 10^7^ cells, resuspended in 200 *μ*l FBS-free culture medium were injected subcutaneously into the right flanks of the mice. The tumor volume was directly measured following inoculation and weight calculated using the formula: (length × width^2^)/2.

### RNA-binding protein immunoprecipitation assay

The RIP assay was performed using the Magna RIP RNA-Binding Protein Immunoprecipitation Kit (Millipore, Bedford, MA, USA) following the manufacturer’s protocol. Briefly, cells at 80–90% confluency were collected and lysed using RIP lysis buffer. One hundred microliters of cell extract was then incubated with RIP buffer containing magnetic beads conjugated to human anti-ATG7 antibody or negative control normal mouse IgG. The samples were incubated with Proteinase K to digest the protein and then the immunoprecipitated RNA was isolated. The purified RNA was used for qRT-PCR analysis.

### Immunohistochemistry

Paraffin-embedded tissue sections were deparaffinized in xylene and rehydrated in a graded series of ethanol solutions and then incubated for 20 min in 3% H_2_O_2_ to quench the endogenous peroxidase activity. Next, the sections were heated in target retrieval solution (Dako, Carpinteria, CA, USA) for 15 min in a microwave oven (Oriental Rotor, Tokyo, Japan) to retrieve the antigen. Nonspecific binding was blocked by incubating with 10% goat serum for 2 h at room temperature. The slides were then incubated overnight at 4 °C with anti-ATG7, LC3, ITGB1 or SQSTM1 (1:100) primary antibody, after which secondary antibody was added and incubated for 20 min at 37 °C, and the binding was visualized with 3, 39-diaminobenzidine tetrahydrochloride. After each treatment, the slides were washed three times with TBST for 5 min.

### IF staining

IF staining was performed according to the standard protocol (Santa Cruz Biotechnology). Briefly, a 5-*μ*m frozen section from each sample was fixed in acetone at 4 °C overnight. After washing with PBS three times, sections were blocked with 1% bovine serum albumin for 30 min and incubated overnight at 4 °C with rabbit anti-human LC3, SQSTM1, ATG7 or LAMP1 primary antibody (1 : 50). Following PBS washes, the sections were incubated with anti-rabbit IgG–TRITC (1 : 100, Santa Cruz Biotechnology) for 2 h at room temperature in the dark, and then washed again with PBS. Nuclei were stained with diamino-phenyl-indole (DAPI, 1 *μ*g/ml; Sigma-Aldrich, St Louis, MO, USA) for 5 min at room temperature. Coverslips were mounted with SlowFade Gold Antifade Reagent (Invitrogen, Carlsbad, CA, USA), and all sections were imaged using a laser confocal microscope (Olympus, Tokyo, Japan).

### Statistical analyses

Statistical analyses were performed using SPSS 17.0 (SPSS, Chicago, IL, USA). Spearman’s correlation test was used to analyze the rank data and a Mann–Whitney *U*-test used to differentiate the means of different groups. Kaplan–Meier survival plots were generated and comparisons were constructed with log-rank statistics. All data are shown as mean±S.D. from at least three separate experiments. *P*<0.05 was considered statistically significant.

## Publisher’s Note

Springer Nature remains neutral with regard to jurisdictional claims in published maps and institutional affiliations.
