# Supplementary material for: The lncRNA HULC functions as an oncogene by targeting ATG7 and ITGB1 in epithelial ovarian carcinoma
Source: Cell Death Dis. 2017 Oct 12;8(10):e3118–. doi: 10.1038/cddis.2017.486 (PMC5682654; doi:10.1038/cddis.2017.486)
Supplement: Supplementary Table 1 [file cddis2017486x1.doc]

**Supplementary Table 1:** Primers for RT-PCR

| **Gene** | **Primer sequence** | **Product size (bp)** | **Extension time (sec)** |
| --- | --- | --- | --- |
| *HULC* | F: 5'- CTGGCAATAAACTAAGCA-3'  R: 5'- CAACATAATTCAGGGAGAA-3' | 94 | 34 |
| *ATG7* | F: 5'- GAACAAGCAGCAAATGA-3'  R: 5'- GACAGAGGGCAGGATAG-3' | 148 | 34 |
| *LC3* | F: 5’- TCGCCGACCGCTGTAA-3’ | 286 | 34 |
|  | R: 5’- AAGCCGTCCTCGTCTTTCT-3’ |  |  |
| *SQSTM1* | F: 5’- TGGAGCACGGAGGGAA-3’ | 304 | 34 |
|  | R: 5’- TCTGGCATCTGTAGGGACTG-3’ |  |  |
| *LAMP1* | F: 5’- TGACAAGGCTTCTCAACATC-3’ | 126 | 34 |
|  | R: 5’- CATTCATCCCGAACTGG-3’ |  |  |
| *18s* | F: 5’- GAAACGGCTACCACATCC-3’ | 167 | 34 |
|  | R: 5’- ACCAGACTTGCCCTCCA-3’ |  |  |

AT = annealing temperature
